# The Application and Outcome Evaluation of a Social Marketing Intervention to Increase Seasonal Influenza Vaccination among University Students

**DOI:** 10.3390/vaccines10101671

**Published:** 2022-10-07

**Authors:** Daisy Lee, Sharyn Rundle-Thiele, Ben Y. F. Fong, Gabriel Li

**Affiliations:** 1 School of Professional Education and Executive Development, The Hong Kong Polytechnic University, Hong Kong, China; 2 Social Marketing @ Griffith, Griffith University, Nathan, QLD 4111, Australia

**Keywords:** seasonal influenza vaccination, social marketing, co-create-build-engage (CBE) framework, college students, university students, behaviour change, outcome evaluation, health promotion

## Abstract

Seasonal flu vaccination rates among university students are exceedingly low and research focused on voluntarily influencing vaccination uptake is limited. This study outlines the development, implementation, and evaluation of a theory-driven social marketing vaccination intervention following the co-create-build-engage (CBE) framework. This study drew upon a pre-intervention segmentation study and co-created interventions targeted at receptive segments. The intervention delivered a significant 343% increase in vaccination rates using a difference-in-difference analysis. Online communication channels effectively engaged students to register for and receive their flu vaccine Almost 90% of students who received vaccinations signed up in the first two weeks of the intervention program indicating that those who can be motivated will act early in the flu season. Financial incentives, as found in previous studies, were confirmed as highly effective in increasing awareness and influencing vaccination uptake behaviours. Friend referral incentives were also found to be influential in motivating vaccination uptake. Suggestions are provided for future research and practical implementation of interventions on university campuses to motivate flu vaccination and other health behaviours.

## 1. Introduction

Seasonal influenza has been a threat to human health for centuries. It is estimated that seasonal influenza might have caused over 400,000 respiratory deaths a year on average [1]. Scientific and technological breakthroughs at the beginning of the 20th century led to the creation of the first influenza vaccine in the 1930s, and vaccination remains the most effective way to prevent influenza and subsequent illnesses and deaths [2,3]. Promoting vaccination against seasonal influenza among the whole population is therefore critical to prevent the spread of influenza. If vaccination coverage could reach 30% or above, the transmission of influenza viruses could be blocked within the community, thus achieving herd immunity [4].

A realistic approach to promoting influenza vaccination might be prioritising resources focusing on vaccination among high-risk groups (i.e., adults aged 65 years or older, pregnant women, children etc.). Subsets of the population with low influenza vaccination uptake, namely university and college students, are often overlooked despite reports around the world warning of their low vaccine uptake [5,6,7]. To achieve herd immunity, promoting vaccination against seasonal influenza among university and college students may be as important as among other population segments if herd immunity is to be achieved. 

A systematic review and meta-analysis conducted by Lee et al. [8] identified that there are only 12 peer-reviewed studies that have focused on voluntary behavioural interventions targeting university or college students. Out of 5291 scholarly works available in major health and academic research databases concerning influenza vaccination promotion, 2836 pieces of literature focused on non-behavioural outcomes such as attitude and intention and 2455 papers studied target groups other than university students (e.g., healthcare workers and high-risk populations such as young children, pregnant women, patients with chronic illnesses, and elderly people). However, university and college students warrant attention. Recognising that morbidity in students associated with seasonal influenza could interrupt students’ socialisation and diminish their academic and workplace performance, various scholars have been attempting to explore means to promote vaccination uptake in college or university student populations [9,10,11]. Moreover, among all peer-reviewed studies on interventions that aimed to increase university students’ uptake of the influenza vaccine, almost all studies were conducted in the USA with only one in Australia and one in Spain [8]. Thus, there exists a huge research gap in understanding effective approaches that can be applied in countries other than the United States to understand whether university students may be voluntarily influenced to receive seasonal flu vaccines.

In response to the lack of studies on voluntary behaviour change interventions to improve seasonal influenza vaccination rates among university students in countries other than the USA, this study was intended to develop and evaluate the effectiveness of a voluntary flu vaccination programme in Hong Kong. This study applied a social marketing approach to influence vaccination behaviour. Despite World Health Organization [12] recommendations to implement social marketing, none of the prior peer-reviewed influenza behaviour intervention studies [8] have comprehensively applied theory-based social marketing. WHO’s SAGE Working Group along with other scholars note social marketing’s capacity to voluntarily motivate behaviour change through effectively communicating and ensuring the delivery of value for vaccinations in a way that is relevant to different audience segments [13]. Hence, this flu vaccination programme followed Rundle-Thiele et al.’s co-create-build-engage (CBE) framework [14] to co-design a social marketing programme with university students and implement the programme on campus to motivate the target vaccine recipient group(s) to voluntarily receive a seasonal flu vaccine. Having the strategies verified with the students themselves, this study then implemented a social marketing intervention programme in a Hong Kong university during the 2021–22 flu season, resulting in actual behaviour change in vaccinations among the target audience. 

To fill the research gap on the lack of seasonal influenza vaccination interventions that applied a social marketing approach to increase vaccine uptake among university students, this study aims to (i) demonstrate how the social marketing programme was developed using the CBE framework to promote vaccination behaviour; (ii) evaluate outcome performance of the social marketing programme implemented; and (iii) provide insights to inform health policy and health promotion that contribute to effective seasonal influenza vaccination interventions.

## 2. Materials and Methods

### 2.1. Study Design

This seasonal influenza vaccination study was conducted at a local university in Hong Kong with three campuses during the 2021–22 flu season (September 2021 to March 2022). The intervention programme was conducted campus-wide in the self-financed unit of the university with 9533 undergraduate students located on two campuses. The third campus offering government-funded tertiary university programs with 15,151 undergraduate students was taken as the control group. Since there are no cross-campus activities between the intervention campuses and the control campus, students in the control campus were not exposed to any intervention materials throughout the research period. All three campuses offer similar disciplines of programs except that the control campus also offers health programs (e.g., nursing, physiotherapy, and occupational therapy) in which students will usually be required to receive flu vaccine prior to clinical internship. Since social marketing studies focus to influence ‘voluntary’ behaviour, the campus with health programs was used as the control campus.

In previous years, all three campuses have promoted seasonal flu vaccination using the same promotion activities. In the intervention year, additional promotional materials developed for this study would only be presented in two intervention campus groups. Usual promotion tactics that were used in previous years were also delivered across all campuses (ie., both intervention campuses and the control campus). Besides comparison with the control group, flu vaccine uptake during the 2021–2022 season would also be compared with data from the 2018–2019, 2019–2020, and 2020–2021 seasons as a baseline measure. This is to ensure that the reference point to which the programme’s effectiveness was compared would not be affected by external and environmental factors, such as the 2019 social movement and the COVID-19 pandemic that caused campus close-downs and suspension of on-campus health centre services. 

### 2.2. Intervention Programme Design Following the CBE Framework

**Figure 1:**
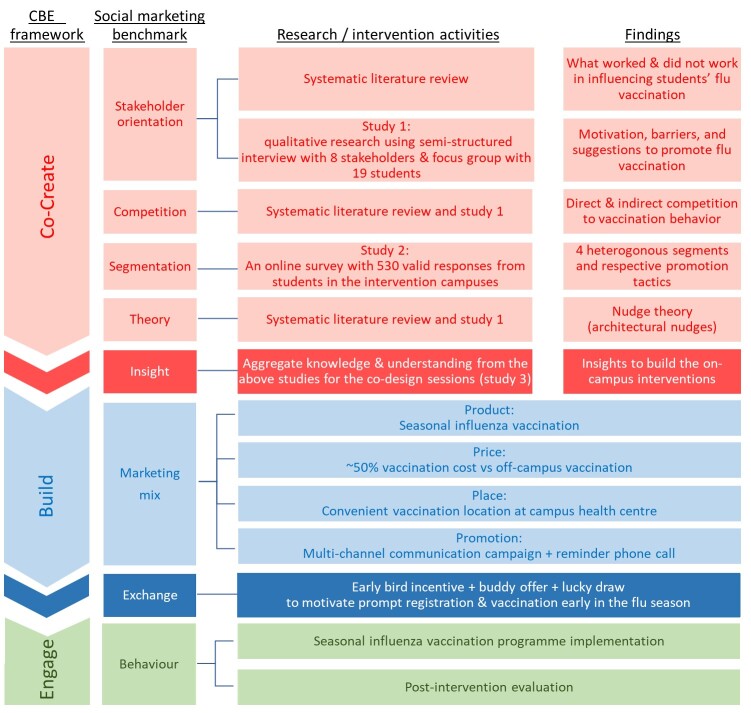
Illustration of intervention design following the CBE framework.

The flu vaccination social marketing programme in this study was developed, implemented, and evaluated following the co-create-build-engage (CBE) framework [14]. The social marketing benchmark criteria were firstly proposed by Andreasen [15] and were further expanded into the eight benchmark criteria by the National Social Marketing Centre [16], these were supposed to serve as the principles for social marketers to qualify their interventions as social marketing. Realising there is still little effort made toward applying the principles which set social marketing apart from other behavioural science approaches, Rundle-Thiele et al. [14] developed the CBE framework to serve as a three-step guide for the design, implementation, and evaluation of a genuine social marketing programme. Founded on top of the social marketing benchmark criteria, the CBE framework provides those who would like to design and implement a social marketing programme with more than just an overall checklist delivering a step-by-step guide with instructions on exactly when the eight elements from the benchmark criteria should be involved during the whole process of developing an intervention for the first time. The following sections (Section 2.2.1, Section 2.2.2 and Section 2.2.3) delineate how the social marketing flu vaccination programme in this study was developed and implemented with the guidance of the CBE process. Figure 1 visualises the study design.

#### 2.2.1. Co-Creation

The first step of the CBE process is co-creation. As the name co-creation literally implies, the essence of a social marketing campaign is that the programme should be designed “together with” the target group instead of solely by experts or researchers who then feed the planned intervention to the target group. A deep understanding of the target group should be acquired through listening and learning from the people we are targeting and the other concerned parties surrounding the target audience group [14]. This listening and learning process involves four of the eight social marketing benchmark criteria: stakeholder orientation, competition, segmentation, and theory. The understanding gained from this process generates insights for the programme design, fulfilling the fifth benchmark criteria. To achieve the aforementioned, a systematic review of the literature and three studies (study 1—a qualitative study including stakeholder interviews and student focus groups; study 2—a quantitative segmentation study; and study 3—co-design sessions with students) were carried out.

#### Stakeholder Orientation

Upon completion of a systematic review of the literature and meta-analysis of previous flu vaccination programmes on university students, study 1 was developed as a qualitative study including semi-structured interviews with major stakeholders and focus groups with students. An interview protocol was developed based on the systematic literature review [8] to explore the knowledge, attitudes, and behaviours of students towards influenza vaccination, along with possible motivation, barriers, and suggestions for future on-campus flu vaccination promotion. Eight people who were heavily involved in the planning, implementation, and administration of on-campus flu vaccination programs were invited to participate in stakeholder interviews. Interviewees included one health centre director, three staff, two student affairs officers who support the campus health centre to disseminate vaccination promotion materials, and two school managers who oversee health centre service quality. Moreover, 6 online focus groups were conducted with 19 students.

#### Competition

Competition to the flu vaccination behaviour was identified from the systematic literature review and qualitative research in study 1. The direct competition was primarily off-campus flu vaccinations provided by private clinics located near students’ homes which operate with more flexible hours. However, the flu vaccine price at private clinics is usually much more expensive than the on-campus option. The indirect competition was comprised of activities such as exercises that are perceived to enhance one’s immunization and these may be considered as a substitution to vaccination. 

#### Segmentation

Study 2 was a segmentation study. Audience segmentation is one of the critical parts of a social marketing campaign. Instead of presuming students from the same college are homogeneous, segmentation studies identify distinctive subsets and the common characteristics exhibited by each sub-set of students. Each segment demonstrates unique characteristics in terms of flu vaccination attitudes, involvement in decision-making, and behaviour. A divide-and-conquer approach can therefore be applied to tackle vaccine hesitancy in each segment, increasing the effectiveness of a behaviour intervention program. 

Based on the interview with stakeholders (study 1), a self-administered online questionnaire survey was designed. Full-time students in the intervention campuses were recruited to participate through a school mass email featuring a description of the research project and a link to the survey four months before the vaccination programme was implemented on campus. A total of 530 valid responses were obtained for segmentation analysis. Data analysed included their past influenza vaccination behaviour, level of involvement in flu vaccination decisions, seasonal influenza-related information search behaviour, and their attention to influenza-related information. According to this segmentation study [17], four heterogeneous segments were identified: (1) convinced, (2) informed unconvinced, (3) open to persuasion, and (4) disengaged sceptics. The “convinced” segment consists of students who are highly receptive to seasonal influenza vaccine programmes and who were planning to receive flu vaccines in the coming flu season. The “open to persuasion” segment featured students who are indifferent to seasonal influenza and flu vaccination. They do not actively search for influenza-related information, but they still pay high attention when they are exposed to such information. Students among the “informed unconvinced” segment have the highest attention to influenza-related information and are characterised by their active searching of such information but they are not necessarily convinced they should receive vaccinations. Lastly, unlike students from the previous three segments, some students see vaccination as unimportant and unnecessary. They have never received flu vaccinations, they do not search for influenza-related information, and they pay little attention even if they are exposed to such information. These students belong to the “disengaged sceptics” segment. The segmentation study revealed that nearly half of the students are receptive to flu vaccination with “convinced” and “open to persuasion” segments comprising 9% and 40% of the student population, respectively. The remaining 51% of students have strong flu vaccine hesitancy and they may refuse to uptake flu vaccines voluntarily. 

#### Theory

Theory application is a core element in social marketing as it enables social marketers to gain a deeper understanding of their audience and design interventions according to the theory, thereby bridging the intention-behaviour gap in vaccine uptake [18,19]. According to the systematic literature review [8] and qualitative study with students, incentives were indicated as a mechanism that could be used to nudge students to receive flu vaccines. Thus, this flu vaccination intervention programme was developed using the nudge theory. While education nudges are found to be less effective, architectural nudges were embedded in programme design to enhance salience and simplify vaccination decision-making, registration, and behavioural action.

#### Insight

Through the systematic review [8], qualitative, and segmentation studies [17] the following major insights were acquired, which guided the co-design of the social marketing programme with students.

1. The “informed unconvinced” and “disengaged sceptics” held strong disbelief in the necessity of preventing flu complications through vaccination, indicating these groups would not be motivated to receive a flu vaccine by an intervention program.
2. Receptive audiences who are “convinced” or “open to persuasion” usually respond to vaccination programs promptly at the beginning of the flu season as soon as they are exposed or reminded to get vaccinated. Thus, a short one to two months vaccination promotion programme will serve the needs of these segment groups. Interventions that last for a few months are intended to remind those who have not been vaccinated in the first 1 to 2 months to act, and this was indicated as an approach that would have minimal effect on increasing vaccination rates.
3. For the other segments who have stronger vaccine acceptance, architectural nudges including free vaccines, incentives to receive vaccinations, and convenience—including simplifying registration and vaccination processes—would support increases in vaccination rates. Non-monetary nudges were indicated as being ineffective.
4. Multi-channel promotion that exposes students to the vaccination programme at all possible on-campus touch points is needed.
5. Visual design of the promotion communication should cut through the clutter to attract students’ attention as students are bombarded with piles of information across all of the school’s communication channels.
6. Peer and parental influence work better than healthcare professionals in influencing vaccination decisions.
7. Post-registration reminders are needed to reinforce vaccination behaviour.
8. While students are exposed to a pile of COVID-19 vaccine information during the pandemic, some students were concerned about the co-administration of COVID-19 and flu vaccines. Hence, the provision of information about the co-administration of these vaccines is required. Communication channels such as the university website, learning portal, and emails are expected to be more effective in reaching students.

Although these insights enable the research team to design an effective vaccination programme, a great uncertainty will remain regarding whether the interventions could effectively engage the students if the programme is solely designed by researchers. Co-design has therefore been employed in this study to involve individuals from the target audience in the designing of strategies and interventions which would be endorsed by the students themselves. Co-design ensured programme design goes beyond the researchers’ limited imagination and incorporates the students’ innovative ideas.

Considering the fundamental differences in the needs and wants of students between different segments [17], not a one-size-fits-all approach but a divide-and-conquer approach could maximise the size of audience that our vaccination programme could effectively engage with. According to insight #1, our programme targeted segments who are receptive to vaccination, aiming to remind and reinforce intention and activate vaccination behaviour. Student segments who are “disengaged sceptics” about flu vaccination were excluded. Our segmentation study has found that most students from this segment have expressed no intention to be vaccinated for the coming season and would not be motivated to do otherwise by any of the tested messages [17]. In addition, previous flu vaccination interventions have found that vaccination rates could be significantly increased by closing the intention–behaviour gap among those who are already willing to be vaccinated [8]. Consequently, the strategy of this social marketing programme prioritized promotion efforts across three segments to normalise vaccination among them so that the “disengaged sceptics” might consider vaccination in future intervention periods as vaccine uptake is more normalised.

Two co-design sessions were conducted with students recruited from the intervention campuses. A total of 15 students were recruited, composed of 8 male and 7 female students. The first co-design session consisted of 10 students belonging to the “low involvement, open to persuasion” segment, which was the largest segment identified from the segmentation study [17]. The second session consisted of 3 students from the “convinced” segment and 2 from the “informed unconvinced” segment. Flu vaccination is considered a high involvement decision among these two segments. Each co-design session lasted for 1.5 h, consisting of four parts. In the first two parts, students were asked to write down their barriers to vaccination and to identify what could motivate them to be vaccinated, respectively. In the third part, students were invited to break into smaller groups to brainstorm a few promotion messages among themselves, and then design the key promotion visuals. Afterwards, they voted for the most appealing one among the items their fellow schoolmates have created. Next, students brainstormed a few email subjects for the vaccination programme before they voted for the most eye-catching one. 

The insights generated in the “co-create” step would become the backbone for the next step “build” of the CBE process.

#### 2.2.2. Build

**Figure 2:**
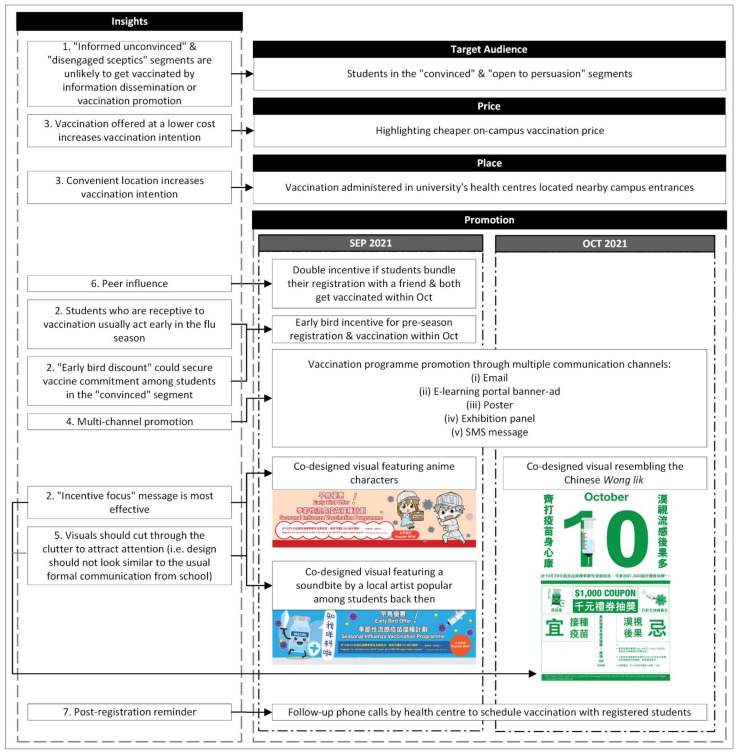
Illustration of how the marketing-mix was built based on insights. Remarks: The numbers shown in front of each insight refer to the insights summarized in Section Insight above.

In the second step of the CBE process, the social marketing programme is built based on the insights gained previously and applies the sixth social marketing benchmark criteria—marketing mix. Commonly known as the “4 Ps” in commercial marketing, the marketing mix is composed of product, price, place, and promotion. Social marketing is more than simply communicating to motivate people. When applied to its fullest extent social marketing seeks to build a new solution which takes account of all the elements in the marketing mix offering additional choices to people [14]. The offered choice should be presented to the people such that the desired behaviour has higher perceived benefits than costs. In that case, exchange (the seventh social marketing benchmark criteria) would take place. Figure 2 illustrates how the marketing-mix in this vaccination programme was built from the insights identified and listed in Section Insight above.

#### Marketing-Mix: Product

The product in this social marketing vaccination programme is the seasonal influenza vaccine. Based on the insights identified, a vaccine from a trustworthy country of origin is crucial even to those who are receptive to flu vaccination. Despite the availability of a newer administration method (i.e., intranasal administration), this campaign would only provide intramuscular injection following the university’s practice. 

#### Marketing-Mix: Price

The vaccination service is provided through the on-campus health centres. The service is subsidised by the university so the vaccination price for students would be 50% less than off-campus vaccination. However, students reported in the focus groups that they were not aware of the lower price of vaccination services on campus. According to insight #3, the cost of the flu vaccine is an important factor influencing vaccination decisions. The programme prominently highlighted the price information on communications. 

#### Marketing-Mix: Place

Within both intervention campuses, the health centres are located in a prominent location easily accessed by students en route to other campus facilities. According to insight #3, it is found that studies succeeded in promoting vaccination uptake by providing the service in easily accessible locations where the target group could conveniently visit (e.g., mobile clinics). The location where students could receive their vaccination in this programme should therefore be convenient enough as they could always visit the health centre on their way to class, before they left, or whenever they are free between classes. Moreover, students will prioritize other school activities over vaccination so the availability of vaccination services at a time convenient to students is also detrimental. In this programme, health centres will pre-arrange a timeslot convenient to the students while they call and confirm the vaccination service with those who have registered. 

#### Marketing-Mix: Promotion

According to insight #4, the programme organized a multi-channel promotion campaign conducted in two phases. The first phase of the programme was an early bird promotion available from late September to early October in 2021, intended to motivate those who have high vaccine acceptance to act. The second phase would be a lucky draw programme starting from the week after the early bird campaign till early November, aimed to influence other receptive audiences to be vaccinated. 

**Communication channels** used to reach the students included (i) email; (ii) banner ads on the landing page of the e-learning portal (e.g., Moodle) which students access daily; (iii) posters displayed in campus lifts or on notice boards located in major hallways in campus; (iv) full-height exhibition panels near major access points of the campus; and (v) SMS broadcasted to students’ cell phone. Soon after students have registered for vaccination through the programme, their contact information would be shared with the university’s health centre so that the clinic could call them for scheduling and remind them to receive the vaccination.

**Visuals** of the printed and online promotion materials originated from students’ ideas contributed through the co-design sessions. Three designs in total were adopted with two of them used for the early bird promotion and the remaining one used for promoting the lucky draw programme. Two visual designs were used to promote the early bird offer. The visual designs are illustrated in this paper. Future studies should replicate the unique propositions behind how visuals were designed: (i) visuals should be eye-catching; and (ii) the look and feel of the visuals should be different from the usual communication materials released by the university; and (iii) visuals should be designed by students from the segment groups targeted. According to insight #5, receptive audiences do not actively search for flu-related information, but they will pay attention to the information when exposed. Therefore, the intervention promotion materials should be designed to cut through communication clutter, and they must be able to attract students’ attention. 

In this study, the first design incorporated two characters from a Japanese anime titled ‘Cells at Work’ which featured anthropomorphised human cells combatting different diseases. At the time when students proposed this visual design, this Japanese manga series was famous around the globe. Thus, the company enabled schools and health organizations to use their visuals royalty free for health promotion [20]. The second design featured an anthropomorphised vaccine bottle with a speech bubble containing a recent soundbite by a local singer and anthropomorphised virus being injected by a syringe. 

Similar to the first phase, the promotional materials used in this phase were co-designed with students. This third design was used to promote the lucky draw and it resembled the traditional Chinese page-a-day calendar. On the posters, exhibition panels, and broadcast emails, the design would contain a big number “10” centred at the upper half of the space to remind students to receive vaccination in October to enter the lucky draw. Big prints of phrases saying lucky draw for thousand-dollar-worth coupons were shown beneath the number “10”. For visuals of promotion materials in high resolution, see Supplementary Materials Figures S1–S3. 

**Messages** on all communication materials adopted an incentive appeal as insight #3 indicated the role of incentives in motivating students’ flu vaccination behaviour. However, past research had indicated that only monetary incentives were effective and that non-monetary nudges had no effect on flu behaviour change among students. This is further confirmed through the segmentation and persona study [17] that indicated incentive focussed messages such as those mentioning early bird offers and emphasising the monetary gain were found to be more persuasive among students from “convinced” and “open to persuasion” segments. According to insight #7, many students are concerned about the interaction between COVID-19 and seasonal flu vaccines, therefore information addressing this concern was emailed to students along with some basic knowledge of seasonal flu vaccines.

#### Exchange

Prior interventions have found that tangible exchange in the form of an incentive is highly effective in maximising the benefits of flu vaccination (insight #3). Moreover, according to insight #6, peer influence is also effective in motivating flu vaccination among students. In this social marketing programme, three types of incentives were utilised to motivate students to engage in vaccination behaviour early in the flu season.

First, an early bird incentive of HKD50 was offered to students who registered for flu vaccination one week before the availability of the flu vaccine. The early bird incentive reduced the actual cost of receiving the vaccine by about 30%. The early bird promotion aimed to secure vaccination commitment among the “convinced” segment of students who have planned to get vaccinated [17]. 

Second, apart from individual registration, students could also register with their friends to obtain an additional buddy offer of HKD50. The early bird and buddy provided a vaccine cost reduction of 55% for each student who registered with a friend within the early bird promotion week. 

Third, throughout October 2021 since the start of seasonal flu vaccination services at campus health centres (the second phase of this programme), students registered to be vaccinated could sign up for a lucky draw. Meanwhile, students who had registered for the early bird offer during the first phase were automatically signed up for the lucky draw. The lucky draw prizes were HKD1000 cash coupons (valued at about 6 times the vaccine cost). 

To successfully bridge the intention-behaviour gap in health behaviour, students would have to receive the flu vaccine at campus health centres by the end of October to be eligible for receiving any of the early bird incentives, buddy offers, and/or lucky draw prizes. According to insight #7, a booking reminder is important to follow through with students from registration to vaccination. 

#### 2.2.3. Engage

The final step of the CBE process was to engage the target group on a full scale with the implementation of the social marketing programme. The awareness of the new solutions built in the previous step has to be broadly raised among the target group and the programme would keep reminding them about the availability of the programme and motivate them to consciously undergo the behaviour change [14].

#### Implementation

While on-campus vaccination services are available throughout the full seasonal flu season (October 2021 to March 2022), this intervention programme was implemented for 5 weeks from the end of September to the end of October 2021. According to insight #2, those who have strong vaccine acceptance will act early to achieve immunity at the beginning of the flu season. This intervention programme was implemented for 5 weeks in two phases:

- Phase 1—one week at the end of September 2021 before the start of vaccine administration as an early bird promotion.
- Phase 2—four weeks in October 2021 from the start date of the vaccination administration service at campus health centres.

To compare the effectiveness of visuals using popular cartoon characters and health-related graphics with popular slang, the following treatment was arranged in phase one. First- and second-year students received an email and were exposed to the Moodle banner ad with the first design for the early bird offer on the first day of the promotion and those with the second design a few days later. Senior years students received emails and were shown the Moodle banner ad with visuals in reverse order. Other promotion materials featuring both designs were displayed throughout the week, including printed A2-size posters in lifts and major hallways where students were passing through frequently, and full-height exhibition panels located at major access points to the campus. SMS containing the text promotion message was also broadcasted to students to remind them of the programme.

During the second phase, promotion materials were sent to the student within the days after the commencement. Throughout the rest of October, promotion materials were visible to students physically on campus as posters and exhibition panels, and virtually as banner ads on the e-learning portal and emails. 

#### Data Collection and Evaluation

This last step of the CBE process is an evaluation of the implemented programme to identify key success factors and key learnings for the enhancement of future programmes to sustain the behaviour change. Besides the actual vaccination rate, the interaction between students and the promotional tools was monitored so that it could be evaluated to learn whether the programme could maximise its reach with the resources available to create the conditions in favour of the desired behaviour change [14]. 

Vaccination numbers were collected from the health centres of both the intervention and control campuses. Health centres also shared the vaccination rate from the previous three years for baseline comparison. QR codes or hyperlinks to access the registration page were assigned to each promotional material, permitting comparisons between communication approaches in the post-campaign evaluation. Students who received their vaccination would be tracked back to see which design successfully motivated them. Similarly, the effectiveness of each promotion channel could also be evaluated. After the intervention programme, students who had participated in either the early bird offer or the lucky draw programme and received vaccination were invited to attend one of the three post-intervention focus groups to understand: (i) students’ unaided recall of promotion materials; (ii) aided awareness of promotion materials; (iii) whether each design had gained their awareness, changed their attitude, intention, and behaviour for vaccination; (iv) why or why not they got vaccinated after registration; and lastly (v) any comments, suggestions, or observations for programme improvement.

## 3. Results

### 3.1. Vaccination Behaviour

**Figure 3:**
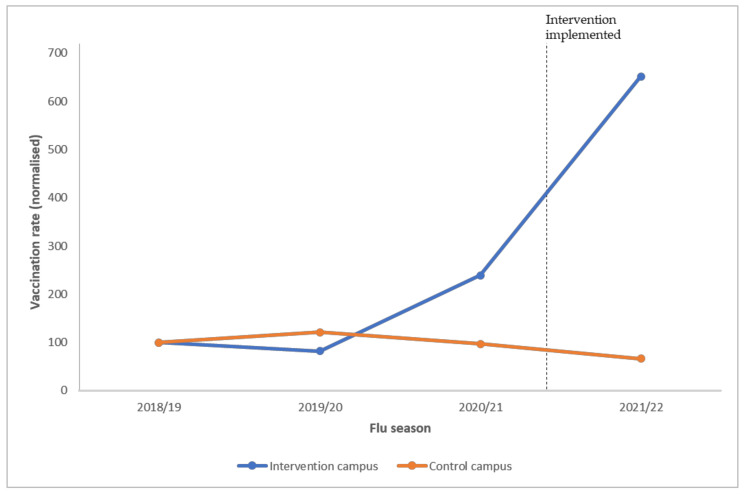
Flu vaccination rate in intervention vs. control campuses. Note: Data for all years are normalised against the 2018/19 season to facilitate comparison.

Vaccination data of both intervention and control campuses were collected through the university health centres from the 2018/19 through 2021/22 flu seasons for a difference-in-difference analysis [21] to compare the intervention and control groups before and after the intervention. This method is commonly used in evaluating changes in health interventions and reduces potential biases from unmeasured social–political factors influencing vaccination rates. For example, during the 2020/21 flu season, people paid more attention to flu vaccination, and they were more willing to take up vaccines as a result of the COVID-19 pandemic. Face-to-face classes were suspended due to the COVID-19 pandemic and all classes were switched to online mode. As students were not physically present on the campus, on-campus vaccination was no longer convenient for students. While trends in the control group approximate what would have happened in the intervention group in the absence of the intervention, the trend will not be mistaken for an intervention effect using the difference-in-difference method. Figure 3 shows the flu vaccination rates in intervention vs. control campuses pre- and post-intervention.

The official vaccination periods ran throughout the winter flu season from 1 October of each year until 31 March of the next year. In previous years, similar vaccination promotion measures were carried out annually on all campuses. In the pre-intervention years of 2018/19 and 2019/20, the vaccination numbers in both the intervention and control campuses remained at similar levels. Although past promotion was repeated in the pre-intervention year of 2020/21, students’ vaccination behaviour was affected by two major forces: (i) the COVID-19 pandemic increased vaccine acceptance; and (ii) the COVID-19 pandemic caused campus close-down and online learning made on-campus vaccination a more inconvenient option for students. The combined effect of these two major forces caused a 191% increase in vaccination rates on the intervention campus but a 20% decrease in the control campus in 2020/21 vs. the 2019/20 season, respectively. During the 2021/22 campaign, the control campus maintained the previous strategy whereas the social marketing interventions co-created with students were implemented on the intervention campuses. The difference-in-difference model shows that the flu vaccination rates on the intervention campus significantly increased by 343% (*p*  <  0.05) vs. pre-intervention, compared to the control group over time. 

Students registered for flu vaccination were categorised into segments according to their answers to questions related to the segmentation variables identified in our former segmentation study [17]. Of all students registered for vaccination, 92% were from segments with high vaccine acceptance (38% from the “convinced” segment and 54% from the “open to persuasion” segment) and only 3% and 5% from the “informed unconvinced” and “disengaged sceptics” segments, respectively. However, only 28% of all registrants finally received the flu vaccine by the end of the October promotion deadline. Although the incentive motivated a large number of registrations among the ‘open to persuasion” segment, it was the “convinced” segment that took up the largest portion of actual vaccinations. The convinced segment represented 52% of the total vaccination count followed by the open to persuasion segment (35%), disengaged sceptics (8%), and informed unconvinced (6%). Chi-square testing indicated a significant difference between segments in vaccination behaviour (χ^2^ (3) = 15.012, *p* < 0.05). Phi and Cramer’s V values were both 0.253, showing a very strong effect of segment difference in vaccination. Reasons for those who did not receive vaccination after voluntarily registering are summarized in Table 1. The top reason is that registrants were not reached by reminder phone calls made by the campus health centre to remind them to schedule their vaccination.

### 3.2. Time to Register and Receive Vaccination

**Figure 4:**
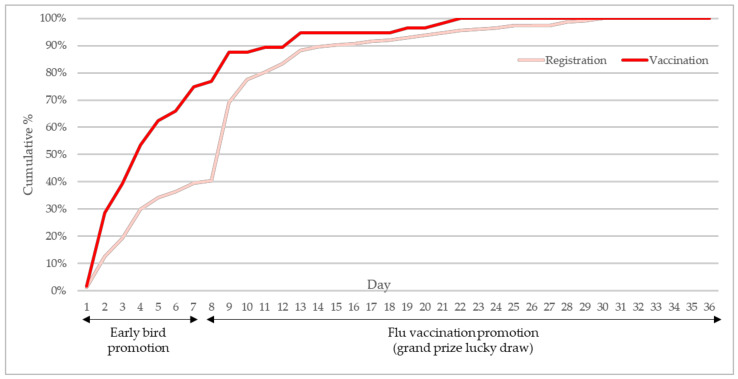
Cumulative percentage of registration by day.

In the intervention year, 91.6% of vaccination uptake throughout the flu season (October 2021 to March 2022) was received in October 2021 during the intervention campaign and only 8.4% were administered in the last five months of the flu season. Figure 4 illustrates the daily cumulative percentage of students who registered through the promotion and finally got vaccinated according to their date of registration. During the campaign period, 39.5% registered in the one-week pre-vaccination season early bird promotion whereas 60.5% registered in the one-month promotion in October. Among those who registered and got vaccinated in October, almost 90% responded to the promotion programme and registered in the first week of October. Moreover, 75.0% of students who finally received vaccination were those registered during the early bird promotion week. 

### 3.3. Effectiveness of Communication Channels and Promotion

**Figure 5:**
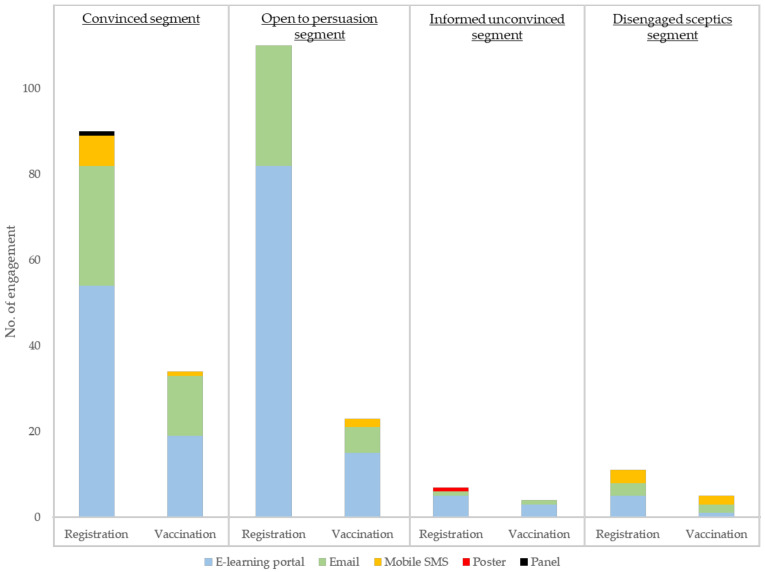
Channel engagement through online and offline channels by student segment.

The effectiveness of communication channels and promotions was examined based on students’ engagement with different marketing communication materials designed by student groups. Engagement data was recorded when students registered using the unique click-through links via email, the e-learning portal, and mobile SMS or QR codes on posters and exhibition panels. Figure 5 shows the number of registrations and vaccinations engaged by each communication channel by segment.

Banner ads on the e-learning portal which students frequently accessed for lecture notes and assignment submissions were the most effective communication channel, sharing 62.1% of total registrations throughout the campaign period. Email was also effective in engaging students to register, contributing to 27.7% of registrations. Mobile SMS also solicited 9.4% of registrations. Only 0.8% of registration were submitted through offline channels (poster and exhibition panel). All registrants who finally received flu vaccination were attracted by online channels (e-learning portal: 34.8%, email: 57.6%, mobile SMS: 7.6%). No significant difference in channel effectiveness was found between segments.

**Figure 6:**
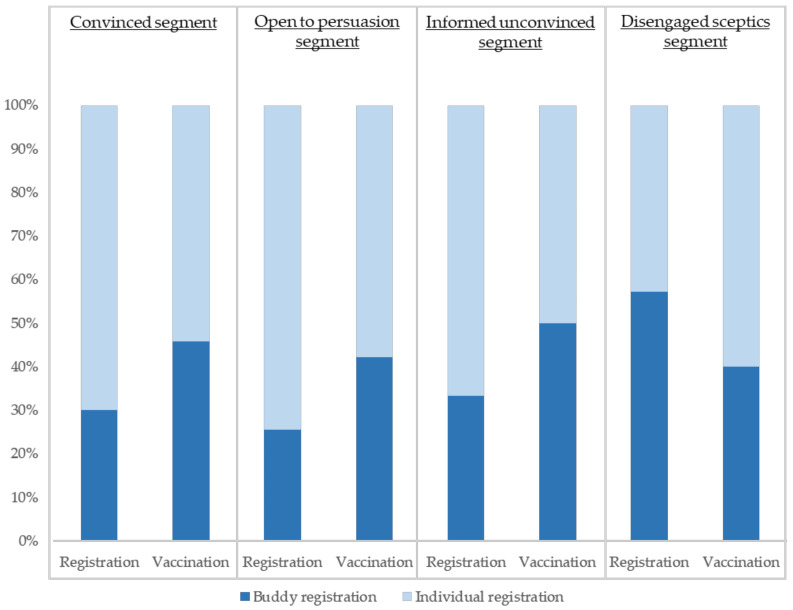
Percentage of registration and vaccination obtained through individual vs. buddy offers by student segment.

During the early bird promotion week, students could register for vaccination individually or with their friends. Students who registered with their friends would get an additional buddy incentive in addition to the individual early bird incentive. The effectiveness of communication channels and promotions was examined based on students’ engagement with the marketing materials. While 30% of registrants signed up for vaccination through the buddy program, 44% of those who were finally vaccinated joined through the buddy program, showing a higher commitment to be vaccinated if students registered with friends. However, this phenomenon was not observed among students from the “disengaged sceptics” segment. Figure 6 shows the percentage of total registrations and vaccinations obtained through individual incentives and the buddy offer for each segment. For convinced, open to persuasion, and informed unconvinced segments, the buddy program represented 30% of registrations and almost 50% of vaccinations. Almost 60% of registrations from students in the disengaged sceptics were due to buddy influence, with 40% electing to receive the flu vaccination with their friends. 

## 4. Discussion

Our study found that a theory-based social marketing intervention co-created with consumers and stakeholders was associated with a substantial increase in on-campus seasonal flu vaccinations among university students. The substantial increase in vaccination rates was facilitated by program co-design following a segmentation study. According to the intervention results, a mass communication program is only effective in motivating students who are receptive to flu vaccination or who are open to persuasion. This finding echoes prior research that a more person-to-person vaccination program is required to motivate seasonal flu vaccination among those with high vaccine hesitancy [22]. 

In motivating vaccinations among students, architectural nudges including free vaccines, incentives to receive vaccinations, and considerations of convenience and simplification of registrations and vaccination processes were found to be effective mechanisms that can be applied to increase vaccination uptake in university populations. Moreover, peer influence not only enhanced students’ intention to receive the flu vaccine, but the buddy offer also encouraged students to mobilise others to get vaccinated. Prior research [19] found that peer endorsement by email did not increase flu vaccination uptake but this study confirmed the effect of offering an incentive to students to “ensure” their friends got vaccinated. However, the buddy system did not work for the disengaged sceptic segments as well as their peers likely did not express any views contradictory to their own and therefore, represent an untapped group. In short, the program should be built to engage students to increase vaccinations instead of simply informing them or aiming to enhance their intention to act. While financial incentives are effective in motivating students to receive vaccinations, it is recommended to prioritise the promotion budget of a buddy offer if there is a budget limitation, to offer both individual and friend-get-friend incentives in future interventions.

Although multi-channel communication was identified as a success factor in previous campaigns, no studies have compared channel effectiveness to aid investment priority [8]. This study found that offline communication channels are barely useful in engaging students in the digital era. Class delivery had moved online during the COVID-19 pandemic limiting foot traffic on campus, which in turn would be expected to diminish offline promotion effectiveness. Almost all vaccination registrations in this intervention study were done via online communication channels, predominantly the e-learning portal and email. Some marketers would argue that although offline channels such as posters and display panels might play a role in enhancing campaign awareness they were not contributing to registrations during the intervention engagement period. Low scan-through rates from QR codes on offline materials and feedback received in post-campaign focus groups, suggested that students were not aware of the offline communication materials. The unaided and aided recall of offline materials was almost zero. Students claimed that they seldom pay attention to wall and lift posters or other display materials on campus because they either talk to friends or browse their phones rather than look around. Thus, banner ads on the e-learning portal were found to be most effective to engage students because they visited the portal every day for course materials and assignment submissions. 

The co-design sessions also revealed that catchy visuals are required for the promotion of flu vaccination. Students receive many emails and online information every day. As they do not pay much attention to flu vaccine information when they are exposed to informational messages, the promotion materials and the promotion mechanics must be highly attractive. A key insight provided by students is that the visuals and designs should not resemble formal or traditional communication materials which look like every other communication material delivered on campus.

Similar to the observation in prior flu vaccination programs [6], people who are receptive to vaccination usually act early in the flu season. It is important to motivate and remind them to act at the beginning of the flu season. Vaccination reminders are confirmed to be important in this study as in previous research [23]. Thus, vaccination intervention programs do not need to be run throughout the flu season or last for a long period of time. An effective program can be run for a few weeks at the beginning of the flu season, but it is crucial to follow intention through to behaviour. In this study, almost 60% of registrants were not reached by reminder phone calls and as a result, many did not get vaccinated. When the campus health centre successfully called to confirm and schedule a vaccination with students, uptake rates were highest. When they finished their classes and called back the health centre at lunch time or in the evening, the health centre was not in service. In the future, universities should consider their reminder channels and co-designing a reminder approach is recommended to ensure contact approaches are relevant to students. 

Stakeholder orientation and collaboration are crucial to the development and implementation of effective on-campus flu vaccination interventions. Careful consideration of the most effective communication channels to influence students is also needed. For example, the banner ad on the most prominent location on the front page of the e-learning portal is an ‘advertising’ space that every department would fight for. Therefore, it requires early engagement with various stakeholders to ensure that students are exposed to flu vaccination communication at the beginning of the flu season.

## 5. Conclusions

While this study confirmed the effectiveness of a social marketing approach to increase on-campus seasonal flu vaccine uptake among university students, there were several limitations. First, while difference-in-difference models enable us to control for underlying variation between pre–post periods among the intervention and control campuses, the groups were not randomly assigned, therefore, unobserved factors may exist. However, this quasi-experiment which was implemented in a real-world setting enhances the generalisability of our results. Second, this intervention study focused on motivating segments with high vaccine acceptance rather than implementing a one-size-fits-all solution. Future intervention studies may explore the feasibility and effectiveness of running multiple programs targeting not only receptive segments but also students with high vaccine hesitancy. Third, this study highlighted the generational differences that we should understand when trying to develop a public health intervention. While this study clearly shows that social media, email, and mobile SMS are very influential for young students, it would be very interesting to apply these same principles to examine if traditional communication channels will be more successful as an intervention for other audience groups, such as the elderly. Fourth, this study focused on motivating the segments with stronger vaccine acceptance to receive the flu vaccine. As students with high vaccine hesitancy comprise about half of the population, future studies focusing particularly on the interventions that are useful for the unconvinced and sceptical groups are needed.

This research is the first study demonstrating how to develop, implement, and evaluate a social marketing intervention using the CBE framework, ensuring the application of all eight principles. Our results suggest that an effective public health intervention program can be co-created with stakeholders and target audiences to effectively influence health behaviours. The steps of the CBE framework in developing a social marketing program can be replicated to deliver practical and powerful interventions capable of addressing a wide range of health issues and agendas among students and the general public.

## Figures and Tables

**Table 1:** Reasons why registrants did not get vaccinated.

Reasons	Convinced (n = 56)	Open to Persuasion (n = 104)	Informed Unconvinced (n = 3)	Disengaged Sceptics (n = 6)	Total (n = 169)
Were not reached by the reminder phone call	80%	86%	67%	67%	83%
No show after phone confirmation	11%	4%	0%	17%	7%
Mistaking the promotion for the COVID-19 vaccine	4%	4%	0%	0%	4%
Cancelled after registration	0%	3%	0%	0%	2%
Did not recall that they had signed up	2%	1%	33%	0%	2%
Too busy to receive vaccination	2%	1%	0%	0%	1%
No longer need the vaccination	0%	1%	0%	0%	1%
Refuse to pay the vaccine cost	0%	0%	0%	17%	1%
Signed up by mistake	0%	1%	0%	0%	1%
Vaccinated off-campus	2%	0%	0%	0%	1%

## Data Availability

The data presented in this study are available on request from the corresponding author.

## Supplementary Materials

The following supporting information can be downloaded at: https://www.mdpi.com/article/10.3390/vaccines10101671/s1, Figure S1: promotion visuals (Japanese anime); Figure S2: promotion visuals (soundbite by a local singer and a syringe); Figure S3 (Chinese calendar).
