# Supplementary material for: The Application and Outcome Evaluation of a Social Marketing Intervention to Increase Seasonal Influenza Vaccination among University Students
Source: Vaccines (Basel). 2022 Oct 7;10(10):1671. doi: 10.3390/vaccines10101671 (PMC9607588; doi:10.3390/vaccines10101671)
Supplement: Supplementary file 1 [file vaccines-10-01671-s001.zip › vaccines-1931833 - supplementary.pdf]

vaccines

MDPI

Supplementary Materials

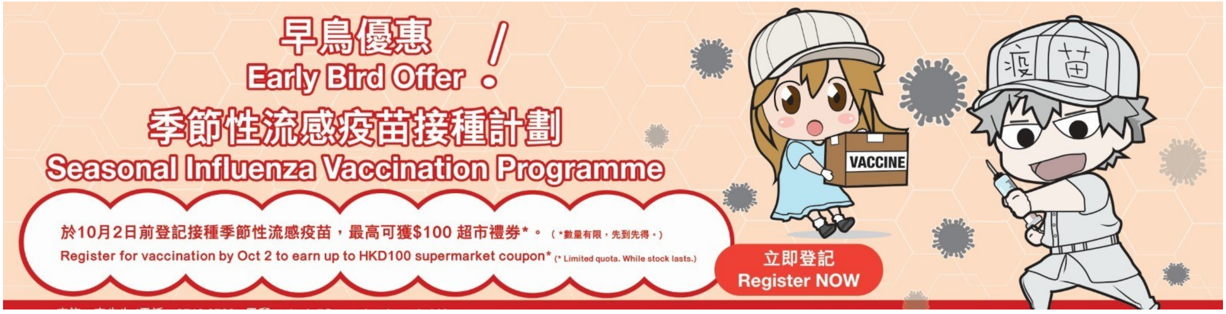

Figure S1. promotion visuals (Japanese anime).

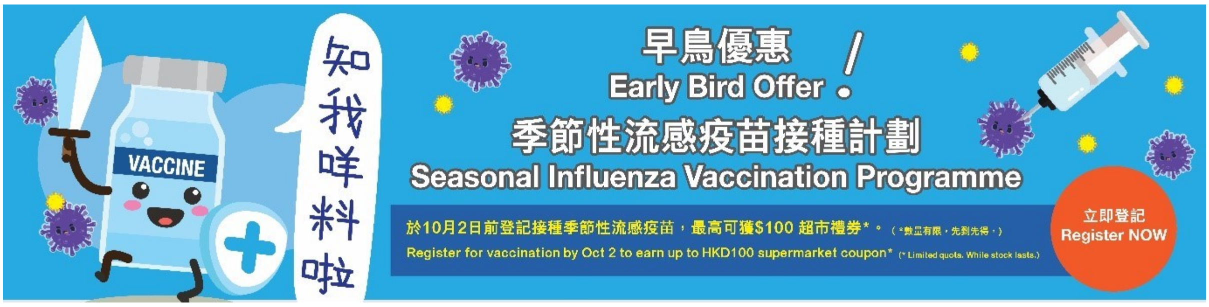

Figure S2. promotion visuals (soundbite by a local singer and a syringe).

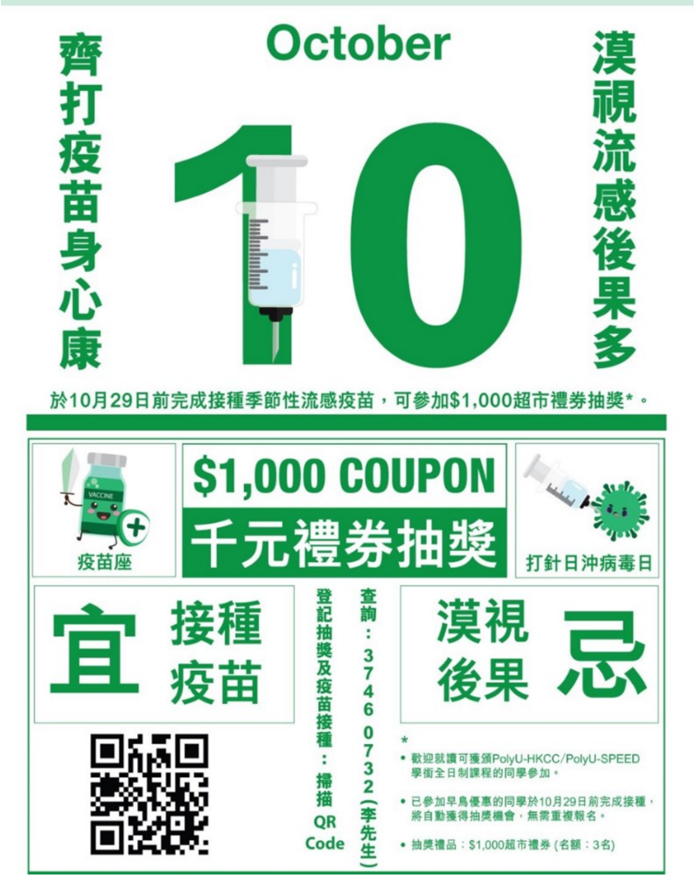

Figure S3. Chinese calendar.
